# Immediate versus expedient emergent laparotomy in unstable isolated abdominal trauma patients

**DOI:** 10.1308/rcsann.2023.0081

**Published:** 2024-06-05

**Authors:** P Maya, B Moran, M Khan, H Yehuda, G Adi, DJ Joseph, Israeli Trauma Group, K Boris

**Affiliations:** ^1^ Schneider Children’s Medical Center, Petah Tikva, Israel; ^2^ Tel Aviv University, Israel; ^3^ Gertner Institute for Epidemiology and Health Policy Research, Tel HaShomer, Israel; ^4^ Brighton and Sussex Medical School, UK; ^5^ Shamir Medical Center, Be’er Ya’akov, Israel; ^6^ University of Texas at Austin, USA; ^7^ Hillel Yaffe Medical Center, Hadera, Israel; ^8^ Technion, Haifa, Israel

**Keywords:** Abdominal trauma, Laparotomy, Emergency surgery, Surgery timing, Mortality

## Abstract

**Introduction:**

Unstable abdominal trauma patients should be treated with emergent laparotomy. However, few studies have evaluated the association between time to surgery and survival in these patients. We aimed to assess the influence of time to laparotomy on outcomes in blunt and penetrating unstable abdominal trauma patients.

**Methods:**

This retrospective study includes patients with abdominal injuries, systolic blood pressure <90mmHg on arrival, admitted in Israel during 2000–2018. Data regarding patients’ characteristics, Injury Severity Score (ISS), Glasgow Coma Scale (GCS), time to surgery, length of hospital stay and mortality were collected via The Israeli National Trauma Registry.

**Results:**

Overall, 69 blunt and 127 penetrating injury patients were included in the study. For blunt and penetrating trauma patients with ISS ≤14, no differences in outcome were found between patients who underwent laparotomy within 60min of admission and those who underwent laparotomy within 60–120min of admission. In patients with blunt trauma, ISS ≥16, and GCS <15, mortality was higher in the immediate laparotomy group (*p* = 0.004 and 0.049, respectively).

**Conclusions:**

In patients with a penetrating injury, no differences in mortality between immediate and expedient laparotomy were demonstrated. In patients with a blunt injury, with ISS ≥16 and GCS <15, mortality was higher among the immediate laparotomy group.

## Introduction

Intra-abdominal trauma is a common cause of injury, responsible for a significant percentage of all trauma casualties in the US.^1^ Blunt injury, caused by motor vehicle crashes or falls, comprises 80% of all abdominal injuries. Penetrating trauma resulting in injury, caused by firearms or stabs, comprises 20% of all abdominal injuries.^1^ There is wide consensus among trauma surgeons that abdominal trauma patients who are haemodynamically unstable on admission should undergo immediate operative treatment.^2^ In addition, in most cases, unstable abdominal trauma patients are operated upon without any imaging because the time from admission to operative treatment is considered to have a critical effect on clinical outcomes and survival.^3^

Nonoperative treatment or laparoscopic surgery are not considered a component of management when patients are unstable, and are recommended only when treating haemodynamically stable trauma patients.^4,5^ In specific circumstances, depending on physiological parameters, the surgical management of unstable abdominal trauma patients consists of damage control laparotomy.^6^ Consensus regarding the need for urgent operative treatment in unstable trauma patients is based, in part, on the “golden hour” concept that was described during the 1970s, where definitive haemorrhage control is achieved within 1h of the index injury. Management according to this concept was reported to be associated with reduced mortality.^7–9^ Although this notion has been accepted worldwide, few clinical studies have investigated whether shortening the time span from admission to definitive treatment does, indeed, reduce mortality.^10,11^

This study aims to investigate the influence of immediate versus expedient laparotomy in unstable abdominal trauma patients on survival and clinical outcomes. In addition, we aimed to assess whether differences in these aspects exist between blunt and penetrating abdominal injuries.

## Methods

### Study design and data collection

This is a retrospective study that includes haemodynamically unstable trauma patients with an isolated abdominal injury, hospitalised and treated in Israel between 2000 and 2018. Haemodynamic instability was defined as systolic blood pressure (SBP) less than 90mmHg on admission. Only patients with isolated abdominal injury or with concomitant injuries, having Abbreviated Injury Scale (AIS) score of 1–2 were included. Only patients with concomitant injuries with AIS 1–2 were included to focus on patients with isolated or near-isolated abdominal injuries. AIS 1–2 was chosen because such injuries are considered minor and should have little to no impact on management priorities. Patients who arrived at the emergency department (ER) without signs of life or patients with an SBP higher than 90mmHg were excluded from the study.

All data were collected via The Israeli National Trauma Registry (INTR) maintained by Israel’s National Center for Trauma and Emergency Medicine Research, as part of the Gertner Institute for Epidemiology and Health Policy and Research. The INTR records information regarding trauma patients hospitalised in 19 different medical centres across Israel, of which 6 are Level I trauma centres and 13 are Level II trauma centres. All trauma patients with an The International Classification of Diseases, Ninth Revision, Clinical Modification (ICD-9-CM) diagnosis code between 800 and 959.9 are registered, including patients who died in the ER or patients who were transferred to a different hospital. This registry records data for more than 90% of all trauma patients and 98% of all severe trauma patients in Israel. The INTR does not collect data regarding patients who died at the scene or en route to the hospital and patients who were discharged from the ER and were not hospitalised.

Data regarding patient’s age and gender, mechanism of injury, vital signs on admission, Injury Severity Score (ISS), Glasgow Coma Scale (GCS), time to surgery, surgical procedure, length of hospitalisation in an intensive care unit (ICU), total length of hospital stay (LOS) and survival were collected for each patient included in this study. The primary endpoint of the study was mortality and the secondary endpoints were ICU stay, duration of ICU stay and total LOS.

### Definitions

Immediate laparotomy was defined as laparotomy within 60min of hospital admission. This definition was based on the “golden hour” concept of achieving definitive haemorrhage control within 1h of admission. Expedient laparotomy was defined as laparotomy within 60–120min of admission. This time frame for the expedient laparotomy group was chosen to ensure that all patients included in this study did undergo an “emergency laparotomy”. The decision to compare patients who were operated upon within 1h and those operated upon later was based on the “golden hour” concept.^7–9^

### Data analysis

Analysis of all data was performed separately for patients with blunt and penetrating injuries. A comparison between the immediate laparotomy group and the expedient laparotomy group was performed regarding clinical outcomes, including length of hospitalisation in an ICU, total LOS and mortality. In addition, stratification of clinical outcomes was performed for ISS and GCS within each group.

### Statistical analysis

Comparison between the study’s groups was performed for baseline and studied variables. Continuous variables were compared using the Mann–Whitney *U* test. Categorical variables were compared by either the chi-squared test or Fisher’s exact test. Statistical significance was considered as a two-tailed *p*-value of ≤0.05. All analyses were performed using SAS software, version 9.4 (SAS Institute, Cary, NC, USA).

### Ethical approval

This study was approved by the Institutional Review Board of the Sheba Medical Center (Approval No. SMC-18-5138).

## Results

Between 1 January 2000 and 31 December 2018, there were 196 haemodynamically unstable patients with an isolated abdominal injury, who underwent laparotomy within 120min from arrival at the hospital. Of these, 127 (64.8%) patients suffered from penetrating abdominal injury and 69 (35.2%) suffered from blunt abdominal injury.

### Penetrating abdominal injury

We identified 127 trauma patients admitted with isolated penetrating abdominal injuries and SBP <90mmHg on admission. Of these, 119 patients (93.7%) were male and 8 (6.3%) were female. Mean age was 31years. Overall, the mean length of stay in an ICU was 6.7 (±11) days, the mean total LOS was 12 days (±19) and total mortality was 31.5% (40 patients).

In this group, 109 (85.8%) patients underwent immediate laparotomy within 60min of arrival at the hospital (Group A), and 18 (14.2%) patients underwent expedient laparotomy within 60–120min (Group B). A comparison of patients’ characteristics and clinical outcomes is presented in Table 1. No differences regarding gender, age, GCS on admission and ISS were found between patients in the immediate and expedient laparotomy groups. Mean (±SD) LOS in patients who underwent immediate laparotomy and those who underwent expedient laparotomy was 5.9 (±10) and 12 (±3) days, respectively (*p* = 0.008). No other differences in clinical outcomes between patients in groups A and B were found.

**Table 1:** Penetrating abdominal injury

	Immediate laparotomy <60min (*n* = 109)	Expedient laparotomy 60–120min (*n* = 18)	*p*-value
Male, *n* (%)	103 (94.50)	16 (88.89)	0.32
Age (mean ± SD)	30 ± 13	39 ± 18	0.06
GCS <15, *n* (%)	52 (47.71)	5 (27.78)	0.12
ISS, *n* (%)			
≤14	45 (41.28)	7 (38.89)	0.85
≥16	64 (58.72)	11 (61.11)	
ICU stay, *n* (%)	53 (48.62)	8 (44.44)	0.74
LOS in ICU (mean days ± SD)	5.9 ± 10	12 ± 13	**0.008**
Total LOS (mean days ± SD)	12 ± 19	15 ± 17	0.1
Mortality, *n* (%)	37 (33.94)	3 (16.67)	0.14

GCS = Glasgow Coma Scale; ICU = intensive care unit; ISS = Injury Severity Score; LOS = length of stay; min = minutes; SD = standard deviation

### Stratification of clinical outcomes for patients with penetrating injury

Results for stratification of clinical outcomes, including ICU stay, LOS and mortality, according to ISS and GCS are presented in Table 2. In penetrating abdominal injury patients with ISS ≤14, no differences were found with regard to length of stay in an ICU, total LOS, or mortality between patients who underwent immediate laparotomy and those who underwent expedient laparotomy. In patients with an ISS ≥16, length of stay in an ICU for patients in the immediate laparotomy group, and patients in the expedient laparotomy was 6.8 (±11) and 15 (±16) days, respectively (*p* = 0.04). In penetrating abdominal injury patients with GCS <15, no differences in clinical outcomes were found between patients in the immediate and expedient laparotomy groups.

**Table 2:** Stratification of clinical outcomes for patients with penetrating injury, according to ISS and GCS

	Immediate laparotomy <60min	Expedient laparotomy 60–120min	*p*-value
ISS ≤14	*n* = 45	*n* = 7	
ICU stay, *n* (%)	17 (37.78)	3 (42.86)	0.79
LOS in ICU (mean days ± SD)	4.1 ± 7.6	5.3 ± 3.1	0.09
Total LOS (mean days ± SD)	9.4 ± 9.1	9.6 ± 6.2	0.54
Mortality, *n* (%)	5 (11.11)	1 (14.29)	0.81
ISS ≥16	*n* = 64	*n* = 11	
ICU stay, *n* (%)	36 (56.25)	5 (45.45)	0.53
LOS in ICU (mean days ± SD)	6.8 ± 11	15 ± 16	**0.04**
Total LOS (mean days ± SD)	14 ± 24	18 ± 20	0.1
Mortality, *n* (%)	32 (50.00)	2 (18.18)	0.05
GCS = 15	*n* = 57	*n* = 13	
ICU stay, *n* (%)	26 (45.61)	6 (46.15)	0.97
Mortality, *n* (%)	6 (10.53)	1 (7.69)	0.76
GCS <15	*n* = 52	*n* = 5	
ICU stay, *n* (%)	27 (51.92)	2 (40.00)	0.67
Mortality, *n* (%)	31 (59.62)	2 (40.00)	0.64

GCS = Glasgow Coma Scale; ICU = intensive care unit; ISS = Injury Severity Score; LOS = length of stay; min = minutes; SD = standard deviation

### Blunt abdominal injury

We identified 69 patients with an isolated blunt abdominal injury and SBP <90mmHg on admission. Of these, 51 (73.9%) were male and 18 (26.1%) were female. Mean age was 35years. Overall, the mean length of stay in an ICU was 6.9 (±9.4) days, the mean LOS was 8.1 days (±9.9) and mortality was 34.8% (24 patients).

In this group, 40 (58.0%) patients underwent immediate laparotomy (Group C) and 29 (42.0%) patients underwent expedient laparotomy (Group D). A comparison of patients’ demographics, GCS, ISS and clinical outcomes is presented in Table 3. The rate of GCS <15 in the immediate laparotomy group and expedient laparotomy group was 60.0% and 21.0%, respectively (*p* = 0.001). No other significant differences regarding gender, age and ISS were found between the immediate and expedient laparotomy groups. Total LOS was 7 (±9.2) days in the immediate laparotomy group and 9.6 (±11) in the expedient laparotomy group (*p* = 0.022). The mortality rate was 50.0% and 13.8% in the immediate and expedient laparotomy groups, respectively (*p* = 0.002).

**Table 3:** Blunt abdominal injury

	Immediate laparotomy <60min (*n* = 40)	Expedient laparotomy 60–120min (*n* = 29)	*p*-value
Male, *n* (%)	28 (70.00)	23 (79.31)	0.39
Age (mean ± SD)	34 ± 20	37 ± 20	0.64
GCS <15, *n* (%)	24 (60.0)	6 (20.69)	**0.001**
ISS, *n* (%)			
≤14	7 (17.50)	9 (31.03)	0.19
≥16	33 (82.5)	20 (69.0)	
ICU stay, *n* (%)	18 (45.0)	13 (44.83)	0.99
LOS in ICU (mean days ± SD)	5.7 ± 7	8.5 ± 12	0.53
Total LOS (mean days ± SD)	7 ± 9.2	9.6 ± 11	**0.022**
Mortality, *n* (%)	20 (50.00)	4 (13.79)	**0.002**

GCS = Glasgow Coma Scale; ICU = intensive care unit; ISS = Injury Severity Score; LOS = length of stay; min = minutes; SD = standard deviation

### Stratification of clinical outcomes for patients with blunt injury

Results for stratification of clinical outcomes, including ICU stay, LOS and mortality, according to ISS and GCS are presented in Table 4. In blunt abdominal injury patients with an ISS ≤14, no differences were found with regard to length of stay in an ICU unit, total LOS or mortality between patients in the immediate and expedient laparotomy groups. In patients with an ISS ≥16, no differences were found with regard to length of stay in an ICU. Total LOS in patients with an ISS ≥16 who underwent immediate laparotomy, and patients who underwent expedient laparotomy, was 6.2 (±9.5) and 10 (±12) days, respectively (*p* = 0.01). The mortality rates of patients in the immediate and expedient laparotomy groups were 60.6% and 20.0%, respectively (*p* = 0.004). In blunt abdominal injury patients with GCS <15, mortality rates were 79.2% and 33.3% in the immediate and expedient laparotomy groups, respectively (*p* = 0.049).

**Table 4:** Stratification of clinical outcomes for patients with blunt injury, according to ISS and GCS

	Immediate laparotomy <60min	Expedient laparotomy 60–120min	*p*-value
ISS ≤14	*n* = 7	*n* = 9	
ICU stay, *n* (%)	3 (42.86)	1 (11.11)	0.26
LOS in ICU (mean days ± SD)	2.3 ± 1.5	5 ± 0	0.37
Total LOS (mean days ± SD)	11 ± 6.8	8.6 ± 5.8	0.55
Mortality, *n* (%)	0 (0)	0 (0)	
ISS ≥16	*n* = 33	*n* = 20	
ICU stay, *n* (%)	15 (45.45)	12 (60.00)	0.30
LOS in ICU (mean days ± SD)	6.4 ± 7.5	8.8 ± 13	0.76
Total LOS (mean days ± SD)	6.2 ± 9.5)	10 ± 12	**0.01**
Mortality, *n* (%)	20 (60.61)	4 (20.0)	**0.00**
GCS = 15	*n* = 16	*n* = 23	
ICU stay, *n* (%)	10 (62.5)	9 (39.13)	0.15
Mortality, *n* (%)	1 (6.25)	2 (8.70)	0.78
GCS<15	*n* = 24	*n* = 6	
ICU stay, *n* (%)	8 (33.33)	4 (66.67)	0.18
Mortality, *n* (%)	19 (79.17)	2 (33.33)	**0.05**

GCS = Glasgow Coma Scale; ICU = intensive care unit; ISS = Injury Severity Score; LOS = length of stay; min = minutes; SD = standard deviation

## Discussion

Abdominal trauma continues to represent a major treatment challenge in trauma centres around the world and has remained associated with significant morbidity and mortality. In this study, we aimed to evaluate the association between the timing of laparotomy and clinical outcomes in haemodynamically unstable penetrating and blunt abdominal trauma patients. Without any doubt, these patients require urgent surgery. This statement is based on evidence that early bleeding control decreases the incidence of the physiological lethal triad, blood transfusion requirement and risk of hypoxic brain injury, and therefore reduces postoperative morbidity and mortality.^12^ A recent study by Smith *et al* has demonstrated that in patients managed with damage control resuscitation and damage control surgery, the appearance of the “death triad” predicted mortality only in 16.6% of patients. Thus, the authors concluded that other factors might contribute to in-hospital mortality.^13^ One such factor may be time to surgery.

The real impact of “time to surgery” on patients’ outcomes remains unclear and a debate regarding the true association between time to surgery and clinical outcome still exists. In a study published in 2002, Clarke *et al* reported that a shorter time span from admission to laparotomy was associated with reduced mortality in patients undergoing surgery within 90min of admission.^10^ However, in a more recent study, published in 2020, Okada *et al* demonstrated no association between a shorter time to surgery and reduced mortality rates in unstable abdominal injury trauma patients.^11^ Moreover, when the timing of surgery is discussed, it is not clear what the term “immediate” means. In the Merriam-Webster dictionary, the term “immediate” is defined as “occurring without loss or interval of time”,^14^ whereas expedient is defined as “suitable for achieving a particular end in a given circumstance”.^15^ Owing to obvious ethical limitations, there is a lack of prospective controlled studies on this topic and many questions remain unanswered.

No acceptable uniform definition for haemodynamic instability exists. Most decision-making processes are based solely on clinical judgement, which varies worldwide. Although the “golden hour” concept is applied by most trauma systems, there are studies that do not necessarily support the current recommendations. For example, a study on 3,656 trauma patients has investigated this approach and has shown no association between the transport time to an ER and mortality among injured patients with physiological abnormality in the field.^16^ Other studies evaluating the impact of transport times in trauma patients demonstrate no influence of longer evacuation time on mortality.^17,18^ This may be specifically true in a small country, such as Israel, where transport times from the scene of injury to the designated trauma centre, are significantly shorter than in some US areas.

The possible benefit seen in this study may be because achievement of vascular access and the start of resuscitation were performed more cohesively in patients who arrived later at the operating theatre. Another possible benefit of expedient laparotomy is the option to utilise endovascular and hybrid methods such as embolisation, endografts and resuscitative endovascular occlusion of the aorta.^19^ For example, insertion of an aortic balloon before surgery may enable inflation of the balloon intraoperatively and help prevent haemodynamic deterioration during laparotomy. The possible benefit of emergent laparotomy may be supported by a critique published by Lerner *et al*, which presented detailed support for the “golden hour” concept and did not identify any studies to support this concept.^7^

Conversely, is a delay in surgery justifiable? A previous study demonstrated that time to surgery, up to 2h following hospital admission, had no impact on outcomes in stable trauma patients with penetrating abdominal injury.^20^ The current study focuses on initially unstable patients with an abdominal injury and assessed the association between time to surgery and clinical outcome. In patients with penetrating abdominal injury, even after stratification for ISS, no significant differences in clinical outcomes were found between early and late surgery, including length of ICU stay, total LOS and mortality. The only difference in clinical outcome measure was a longer ICU stay in patients with ISS >16 who underwent expedient laparotomy. In patients with blunt abdominal injury, mortality was higher among patients who were operated upon within 60min from admission. Following stratification for ISS and GCS, in patients with ISS ≥16 as well as in patients with GCS <15, mortality remained higher for patients with blunt injury who were operated upon early.

To the best of our knowledge, this is the first study that examines the impact of time to surgery on both blunt and penetrating injury in unstable patients with isolated abdominal injuries. A possible explanation for this result might be that expedient laparotomy may enable involvement of a senior trauma/vascular/anaesthesia team, better preoperative preparations, including blood bank readiness, better preoperative resuscitation, utilisation of different endovascular and hybrid treatment methods,^19^ and/or a preoperative computerised tomography scan that enables better understanding of the injury prior to surgery.

The results of the current study suggest time may not be the sole and most important factor when treating trauma patients. These results are preliminary but may justify additional investigations. Further studies are needed to evaluate what is more important: time to scalpel only or time to resuscitation en route to scalpel. A large database with a computed real-time recording of physiological parameters and interventions may identify possible patients that could benefit from both approaches. We believe our findings suggest a need for future prospective studies regarding the optimal use of time before laparotomy in unstable abdominal trauma patients.

### Limitations

The present study has several limitations. First, despite the use of a national database, this is a retrospective study with a relatively small sample size. Another limitation is that the trauma registry does not include data regarding specific clinical examination findings, as well as a clear description of clinical judgement and criteria for emergency surgery, response to fluids and resuscitation, haemodynamic instability, indications for surgery, reasons for decreased GCS score and reasons for surgery delay. Thus, patients who were operated within 60min may have been more severely injured in a manner that is not represented by data recorded in the registry. In addition, the potential differences in resuscitation protocols between medical centres in this study must be noted. For these reasons, additional prospective studies on this topic are warranted.

## Conclusions

For patients with penetrating injury, no differences in mortality between immediate and expedient laparotomy were found. For patients with blunt injury overall, as well as for more severely injured patients with a high ISS and a low GCS, mortality was significantly higher among patients who were operated upon immediately compared with those with similar injury severity who were operated upon expediently. Although the time to definitive treatment is an important factor when treating unstable patients, this study carefully suggests that an optimal use of time before surgical treatment may be beneficial and have a potentially positive impact on outcome. Certainly, further studies are needed to reinforce or reject these results.

### The Israeli Trauma Group

A Acker, N Aviran, H Bahouth, A Bar, A Becker, M Ben Ely, D Fadeev, I Grevtsev, I Jeroukhimov, A Kedar, A Korin, A Lerner, M Qarawany, AD Schwarz, W Shomar, D Soffer, M Stein, M Venturero, M Weiss, O Yaslowitz, I Zoarets.
